# Effectiveness of Cotreatment with Kuntai Capsule and Climen for Premature Ovarian Failure: A Meta-Analysis

**DOI:** 10.1155/2020/4367359

**Published:** 2020-02-19

**Authors:** Qianwen Ma, Yong Tan, Genlin Mo

**Affiliations:** ^1^Gynecology Department, Zhenjiang Hospital Affiliated to Nanjing University of Chinese Medicine (Zhenjiang Hospital of Traditional Chinese Medicine), Zhenjiang, China; ^2^Reproductive Medicine Department, Affiliated Hospital of Nanjing University of Chinese Medicine, Nanjing, China; ^3^Advanced Manufacturing Institution, Jiangsu University, Zhenjiang, China

## Abstract

**Objective:**

To compare the treatment efficacy of Kuntai capsule with Climen only in the therapy of premature ovarian failure.

**Methods:**

Randomized controlled trials were electronically retrieved from PubMed, Cochrane Library, Web of science, CBM, CNKI, Wanfang, and Weipu database. In addition, some related papers were manually checked. All papers were assessed according to the Cochrane Handbook for Systematic Reviews of Interventions, and the effective data were analyzed by Revman 5.3 Software.

**Results:**

11 randomized control trials involving 1068 patients were included. Results of meta-analysis showed that E_2_ (estrogen), the total therapeutic effective rate of the group of Kuntai capsule, and hormone were higher than hormone only. The LH (luteinizing hormone), FSH (follicle-stimulating hormone), and Kupperman score of the group of Kuntai capsule and Climen were lower than Climen only.

**Conclusion:**

Available evidence shows that Kuntai capsule with Climen is more effective than Climen in the therapy of premature ovarian failure. Nowadays, the quality of the research studies is low. More large-scaled randomized trials will need to be carried out.

## 1. Introduction

In recent years, with the economic development, social progress, increased mental stress day by day, and the adverse living habits lead to a sharp increase in premature ovarian failure (POF) patients [1, 2]. Premature ovarian failure refers to the ovarian function declining of women before 40 years old, belonging to the polymorphic diseases [3, 4]. The main performance of the patients is low estrogen, reduced leucorrhea, amenorrhea, libido, infertility, and emotional abnormal fluctuations. In addition, the patients also suffer from the elevated level of FSH and LH, reduced E_2_ level, and elevated gonadotropin concentration. It may be clinically related to genetic, immune, congenital absence of enzyme, infection, environment, and iatrogenic factors [2,5–13]. Currently, the main treatment for this disease is hormone therapy, which can improve the patients' hormone levels and relieve symptoms [14–16]. However, long-term use of hormone drugs has great side effects, which can stimulate reproductive organs and increase the risk of endometrial cancer. In recent years, traditional Chinese medicine has been gradually applied in the treatment of premature ovarian failure, and its advantages are gradually highlighted. Some scholars have proposed that Climen combined with traditional Chinese medicine can improve the treatment effect and ovarian function, and at the same time, the side effects are small and the medication safety is good [17–27]. In view of this, the purpose of this study is to conduct a meta-analysis of the current clinical evaluation results of Kuntai capsule combined with Climen for premature ovarian failure and to preliminarily evaluate the advantages and disadvantages of Kuntai capsule combined with Climen in the treatment of premature ovarian failure.

## 2. Methods

### 2.1. Inclusion and Exclusion Criteria

We included randomized controlled trials of Kuntai capsule with Climen compared with Climen in women with premature ovarian failure. We included trials no matter where she came from. We eliminated wrong, incomplete, and repeatable articles. We also eliminated the articles of the experimental group which did not use Kuntai capsule with Climen and the control group which did not use Climen monotherapy.

### 2.2. Intervention and Outcome Indicators

The experimental group was Kuntai capsule with Climen. The control group was Climen only. Outcome indicators included the total therapeutic effective rate, LH, FSH, E_2_ level, Kupperman score, and safety evaluation.

### 2.3. Systematic Search for Evidence

The databases of PubMed, Cochrane Library, Web of science, CBM, CNKI, Wanfang, and Weipu were searched from computer to identify relevant RCTs. We also performed a hand search to identify any other articles. The following search terms were used: Kuntai capsule, Kuntai, hormone, estradiol, Climen, progesterone, artificial period, artificial cycle, and premature ovarian failure. The trials should be selected with no restriction.

### 2.4. Data Extraction and Quality Appraisal of the Evidence

Two independent reviewers extracted the data according to the inclusion criteria. If the two reviewers disagreed, the difference was solved through discussion. If a consensus could not be reached, a third reviewer was consulted. Reviewers evaluated the evidence according to the Cochrane system evaluation member handbook on the quality evaluation criteria of RCT [28]: (1) which random allocation method to choose; (2) whether the trial was allocation concealment; (3) whether the trial used the blinding method; (4) whether there was incomplete data bias; (5) whether there was selective bias; (6) other bias.

### 2.5. Data Analysis

All meta-analyses were done in Revman 5.3 provided by the Cochrane Collaboration. The results were reported as odds ratios (ORs), with 95% confidence interval (95% CI) for dichotomous outcomes, and weighted mean difference (WMD) with 95% CI for continuous outcomes. The chi-square test was used to test heterogeneity across studies. Data were analyzed with a fixed effect model if no statistical heterogeneity was observed (*I*^2^ ≦ 50%). Data were analyzed with a random effect model if statistical heterogeneity was observed (*I*^2^ > 50%). In the presence of heterogeneity, the two researchers checked the data entered and explored the variation by conducting sensitivity analysis. Publication bias was examined by the funnel plot. We used the *Z* (*u*)-test to compute statistics. According to the *Z* (*u*), the statistic *P* was obtained. If *P* < 0.05, there was statistical significance; If *P* > 0.05, there was no statistical significance.

## 3. Results

### 3.1. Included Trials

**Figure 1:**
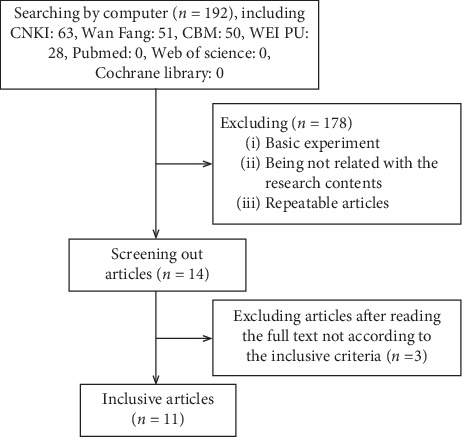
Data screening process.

Searching from each database, we received 192 citations, including 63 in CNKI, 50 in CBM, 51 in Wanfang, 28 in Weipu, 0 in Medline, 0 in Cochrane Library, and 0 in Web of science. Screening the citations, the 178 citations were ruled out (including repeating between libraries and the content which had nothing to do with the study). Then, 14 articles were obtained. By reading the full text, 3 articles were ruled out because they did not meet the inclusion criteria. Finally, 11 articles were included (Figure 1).

### 3.2. Methodological Quality

This study included 11 articles, a total of 1068 patients, which were randomized controlled trials in China. This research showed that there were 10 articles reporting the FSH and E_2_ level. 5 articles reported the LH level. 9 articles reported on the total therapeutic effective rate. 5 articles reported on the Kupperman score. 2 articles reported the safety analysis. See Table 1 for a summary of key details of these studies.

### 3.3. Analysis of the Level of LH

**Figure 2:**
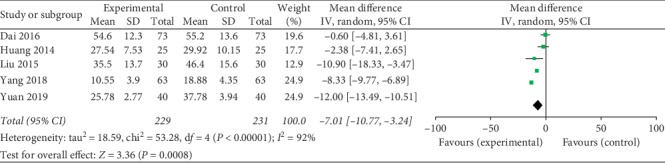
Comparison of the level of LH.

The meta-analysis results showed that the difference among the 5 groups was statistically significant on the level of LH: the combined effect MD = −7.01, 95% CI [−10.77, −3.24], *Z* = 3.65, and *P*=0.0003. So the level of LH of Kuntai capsule with the hormone group was lower than the hormone group (Figure 2).

#### 3.3.1. Sensitivity Analysis

This study analyzed the sensitivity of heterogeneity of the 5 articles. Getting rid of Yuan HF's article decreased the heterogeneity obviously, so it was likely to be the main source of heterogeneity.

### 3.4. Analysis of the Level of FSH

**Figure 3:**
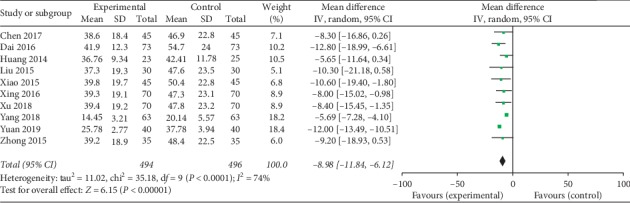
Comparison of the level of FSH.

The meta-analysis results showed that the difference among the 10 groups was statistically significant on the level of FSH: the combined effect MD = −8.98, 95% CI[−11.84, −6.12], *Z* = 6.15, and *P* < 0.00001. So the level of FSH of Kuntai capsule with the hormone group was lower than the hormone group (Figure 3).

#### 3.4.1. Sensitivity Analysis

This study analyzed the sensitivity of heterogeneity of the 10 articles. Getting rid of Yuan HF's article decreased the heterogeneity obviously, so it was likely to be the main source of heterogeneity.

### 3.5. Analysis of the Level of E_2_

**Figure 4:**
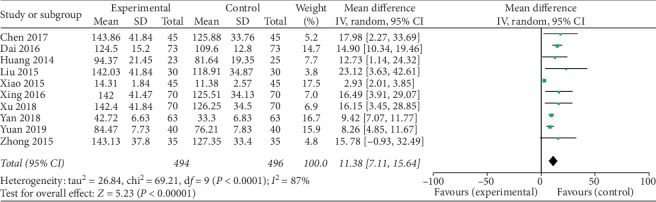
Comparison of the level of E_2_.

The meta-analysis results showed that the difference among the 10 groups was statistically significant on the level of E_2_: the combined effect MD = 11.38, 95% CI [7.11, 15.64], *Z* = 5.23, and *P* < 0.00001. So the level of E_2_ of Kuntai capsule with the hormone group was higher than the hormone group (Figure 4).

#### 3.5.1. Sensitivity Analysis

This study analyzed the sensitivity of heterogeneity of the 10 articles. Getting rid of Xiao's article decreased the heterogeneity obviously, so it was likely to be the main source of heterogeneity.

### 3.6. Analysis of the Therapeutic Effective Rate

**Figure 5:**
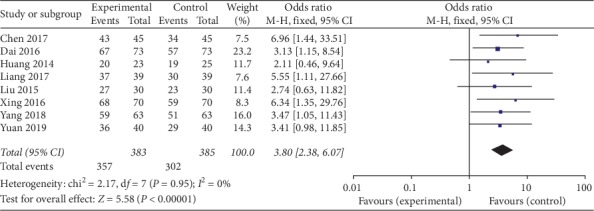
Comparison of the therapeutic effective rate.

The meta-analysis results showed that the difference among the 8 groups was statistically significant on the therapeutic effective rate: the combined effect OR = 3.88, 95% CI [2.47, 6.08], *Z* = 5.90, and *P* < 0.0001. So the therapeutic effective rate of Kuntai capsule with the hormone group was higher than the hormone group (Figure 5).

### 3.7. Analysis of the Kupperman Score

**Figure 6:**
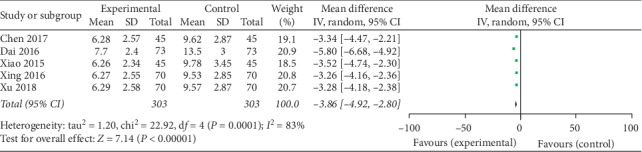
Comparison of the Kupperman score.

The meta-analysis results showed that the difference among the 5 groups was statistically significant on the Kupperman score. The combined effect MD = −3.86, 95% CI [−4.92, −2.80], *Z* = 7.14, *P* < 0.00001. So the Kupperman score of Kuntai capsule with the Climen group was lower than the Climen group (Figure 6).

#### 3.7.1. Sensitivity Analysis

This study analyzed the sensitivity of heterogeneity of the 5 articles. Getting rid of Dai SM's article decreased the heterogeneity obviously, so it was likely to be the main source of heterogeneity.

### 3.8. Safety Analysis and Publication Bias

**Figure 7:**
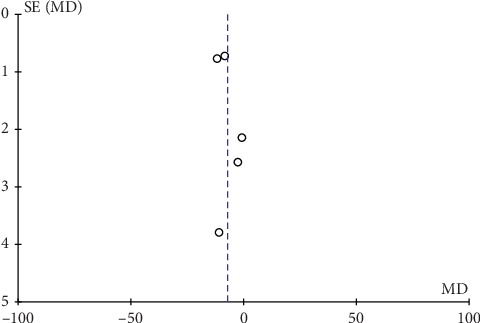
Funnel plot of the level of LH.

**Figure 8:**
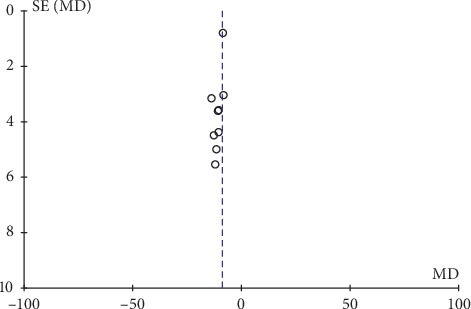
Funnel plot of the level of FSH.

**Figure 9:**
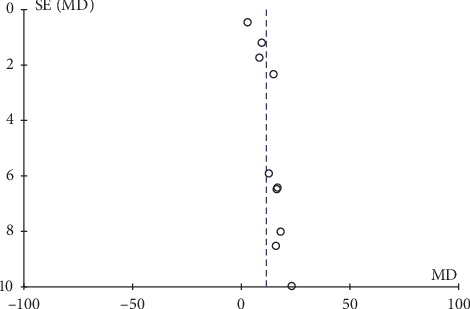
Funnel plot of the level of E_2_.

**Figure 10:**
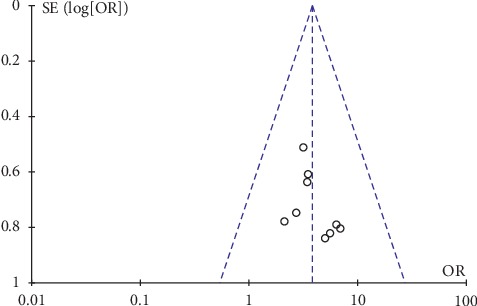
Funnel plot of the total therapeutic effective rate.

**Figure 11:**
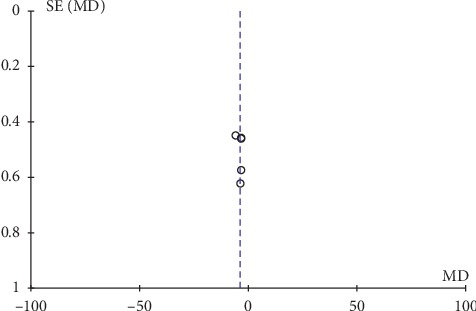
Funnel plot of the Kupperman score.

Only two articles presented adverse effects of their studies, and the descriptions were not very detailed. Thus, we were unable to analyze the safety outcomes. In this study, inverted funnel graph analysis was performed on the inverse of standard error of OR of LH, FSH, E_2_ level, total therapeutic effective rate, and Kupperman score. The asymmetrical pattern suggested small sample studies and possible publication bias, as shown in Figures 7–11.

## 4. Discussion

The results of this study showed that the total therapeutic effective rate, LH, FSH, E_2_ level, Kupperman score of Kuntai capsule, and Climen group were more effective than those of the Climen group in the treatment of premature ovarian failure. POF will not only cause the reduction of estrogen level, but also lead to the loss of female fertility in severe cases, which will exert great pressure on female physiology and psychology and affect the quality of life. Climen [29, 30] (estradiol valerate tablets/estradiol cyprogesterone tablets) is the commonly used Western medicine treatment of POF, which can simulate the sex hormone levels of the menstrual cycle in the body and induce menstrual cramps. But long-term use of Climen may lead to water sodium retention patients and increase the risk of endometrial cancer and breast cancer. Kuntai capsule [31–37] originates from *Rhizoma coptidis* and donkey-hide gelatin soup of Treatise on Febrile and Miscellaneous Disease, mainly composed of *Rhizoma coptidis*, donkey-hide gelatin, radix paeoniae alba, *Poria cocos*, *Radix scutellariae*, and *Rehmannia glutinosa*. It can improve the symptoms such as amenorrhea, hypomenorrhea, bradymenorrhea, night sweats, and lumbar debility. Therefore, Kuntai capsule combined with Climen can combine the advantages of the two therapy methods and make the female reproductive environment more harmonious.

The methodological quality evaluation results of the included researches showed that there were many low-quality studies. The clinical studies included in this study had methodological problems such as randomization, blind method, and follow-up. All of these could lead to the occurrence of bias and affect the accuracy and reliability of the trials. Among the 11 references included in this meta-analysis, only 5 references described the specific randomized method (the random number table method or treatment order), although all the reports mentioned the random method. In most studies, there was no mention of the concealment of the random scheme, and no specific description of the blind method, loss of follow-up, and withdrawal cases, which would affect the demonstration strength of the study. Meanwhile, LH, FSH, E2 level, and total therapeutic effective rate were the main indicators for the evaluation of efficacy, and the safety analysis of drugs and Kupperman score was less concerned. In the future, randomized controlled trials should not only be designed reasonably, but also include large samples, rigorous randomized methods, and double-blind studies.

Therefore, this study considers that the combination of Kuntai capsule and Climen for premature ovarian failure has a better curative effect. However, it needs to be supported by more rigorous double-blind randomized controlled trials with a larger sample to ensure more convinced research results and provide more reliable evidence for the combination of Kuntai capsule and Climen for premature ovarian failure.

## Figures and Tables

**Table 1:** Summary of clinical trials examining the effects of Kuntai capsule with Climen on patients with POF.

Studies	Participants	Treatment group	Control group	Main results	Course of treatment
Xu [17]	*N* = 140	Climen + Kuntai capsule *N* = 70	Climen *N* = 70	Treatment group had a better effect	6 months

Xing [18]	*N* = 140	Climen + Kuntai capsule *N* = 70	Climen *N* = 70	Treatment group had a better effect	6 months

Yang [19]	*N* = 126	Climen + Kuntai capsule *N* = 63	Climen *N* = 63	Treatment group had a better effect	6 months

Yuan and Hu [20]	*N* = 80	Climen + Kuntai capsule *N* = 40	Climen *N* = 40	Treatment group had a better effect	3 months

Chen [21]	*N* = 90	Climen + Kuntai capsule *N* = 45	Climen *N* = 45	Treatment group had a better effect	6 months

Zhong et al. [22]	*N* = 70	Climen + Kuntai capsule *N* = 35	Climen *N* = 35	Treatment group had a better effect	3 months

Dai [23]	*N* = 146	Climen + Kuntai capsule *N* = 73	Climen *N* = 73	Treatment group had a better effect	3 months

Xiao et al. [24]	*N* = 90	Climen + Kuntai capsule *N* = 45	Climen *N* = 45	Treatment group had a better effect	3 months

Liang [25]	*N* = 78	Climen + Kuntai capsule *N* = 39	Climen *N* = 39	Treatment group had a better effect	3 months

Liu [26]	*N* = 60	Climen + Kuntai capsule *N* = 30	Climen *N* = 30	Treatment group had a better effect	6 months

Huang [27]	*N* = 48	Climen + Kuntai capsule *N* = 23	Climen *N* = 25	Treatment group had a better effect	3 weeks
